# A neonate with hand, foot, and mouth disease complicated with brainstem encephalitis and pulmonary edema:A complete recovery

**DOI:** 10.12669/pjms.304.4528

**Published:** 2014

**Authors:** Shi-Jie Guo, Dong-Xuan Wang, Chun-Lai Dai, Hui Wu

**Affiliations:** 1 Shi-Jie Guo, Department of Neonatology, The First Hospital of Jilin University, Changchun 130021, China.; 2 Dong-Xuan Wang, Department of Ultrasonic Diagnosis, The First Hospital of Jilin University, Changchun 130021, China.; 3 Chun-Lai Dai, Department of Radiology, The First Hospital of Jilin University, Changchun 130021, China.; 4 Hui Wu, Department of Neonatology, The First Hospital of Jilin University, Changchun 130021, China.

**Keywords:** Neonate, Hand, Foot and mouth disease, Enterovirus 71, Brainstem encephalitis, Pulmonary edema

## Abstract

Hand, foot, and mouth disease (HFMD) with serious complications and fatal cases have been reported over the last decade worldwide. The authors report a rare case of HFMD in a neonate complicated with brainstem encephalitis and pulmonary edema. She had fever, lethargy, dyspnea. Physical examination revealed shock signs, fine rales on both lungs, absent Moro reflex. The patient had a rapidly progressive course with seizures, coma, no spontaneous breathing, chemosis. There were some vesicles on left sole and red maculopapular rashes on perianal skin. She had a history of exposure to HFMD. Fecal sample was positive for EV71 RNA by real-time PCR. Chest X-rays showed bilateral pulmonary infiltrates. MRI of the brain showed significant hypointensity in the brainstem on T_1_WI and hyperintensity on T_2_WI. She recovered well. This case highlights severe HFMD in neonates is rare. Medical history and physical examination are important in making diagnosis.

## INTRODUCTION

Hand, foot, and mouth disease (HFMD) is a common viral illness caused by enteroviruses that predominantly affect infants and children younger than 5 years old. The prognosis of HFMD is usually good. However, serious complications and fatal cases have been reported over the last decade worldwide. Recently, a fatality rate of 0.4% was reported from Fuyang City, Anhui province in China.^1^ The authors report a rare case of HFMD in a neonate complicated with brainstem encephalitis and pulmonary edema.

## CASE REPORT

A 25-day-old female neonate was admitted to our hospital with fever of three days, lethargy and fast breathing for the past 6 hours. There was history of poor feeding, decreased urine output and pale skin. Maternal history for this baby before and during the disease course was normal. She received breastfeeding. Written informed consent was obtained from participants, and this study was approved by the Institute Review Board of the First Hospital of Jilin University.

Physical examination revealed temperature 38.8ºC, pulse 200/min, respiratory rate 60/min, capillary refill time 6s, lethargy, pallor, mottled skin, pin point miosis, poor pupillary light reflex, feeble pulse, cold extremities, harsh breath sounds, fine rales on both lungs, muffled heart sounds, no murmur and hepatosplenomegaly, hypotonia, absent Moro reflex. 

The laboratory findings were as follows: complete blood count: white blood cell 28.9×10^9^/L, lymphocytes 45%, neutrophils 46%, monocytes 9%, red blood cell 4.27×10^12^/L, hemoglobin 135g/L, platelets 327×10^9^/L; myocardial enzymes: creatine kinase (CK) was 117 U/L, MB isoenzyme of CK 36.7 U/L, lactate dehydrogenase 424 U/L, and normal myocardial troponin; C reactive protein (CRP): normal; blood glucose 27.6 mmol/L; negative blood culture. The chest X-ray showed bilateral pulmonary infiltrates without cardiomegaly and features of pulmonary edema. 

The patient was intubated immediately and put on conventional mechanical ventilation. Blood gas analysis showed severe metabolic acidosis. She was treated with symptomatic, supportive and antibiotic therapies. She had a rapid progressive clinical course with seizures, coma, urinary retention, an absent pupillary light reflex and chemosis. Endotracheal suction revealed sputum. The cerebrospinal fluid (CSF) analysis was normal; however, polymerase chain reaction (PCR) of CSF was not completed. On day 2, a few vesicles on left sole and red maculopapular rashes on perianal skin were found. A review of medical history revealed that she came from a HFMD epidemic area. She was in close contact with her uncle who had a history of exposure to HFMD. Her fecal sample was positive for EV71 RNA by real-time PCR. Bacterial comorbidity was ruled out as the blood culture remained negative, CRP and neutrophil count were normal. HFMD complicated with brainstem encephalitis and pulmonary edema was diagnosed. She was treated in the isolation ward with intravenous high-dose methylprednisolone and immunoglobulin. Her pulmonary rales disappeared, the features of pulmonary edema and acidosis resolved, blood glucose and blood pressure were normal on day 3. Her conscious level improved and she resumed spontaneous respiration on day 4. She was then extubated and supported by nasal continuous positive airway pressure (CPAP). Her brain MRI showed significant hypointensity in the brainstem on T_1_WI and hyperintensity on T_2_WI on day 10. Her consciousness and movement were normalized on day 13. She was able to breastfeed. She was discharged from hospital. She was healthy with normal neurological examinations until she had been followed up for two years.

## DISCUSSION

HFMD typically occurs in small epidemics in nursery schools or kindergarten schools. Spread to other family members commonly occurs. The neonate’s uncle had a history of exposure to HFMD, but he did not have any symptoms. However, she was in close contact with him.

The most common strains to cause HFMD are coxsackievirus A16 (CA16) and enterovirus 71(EV71).^2^ Approximately 50% of neonates are found to have detectable EV71 neutralizing antibodies, which decline to almost undetectable levels by 6 months of age. The lack of protective antibodies in younger children may account for the high incidence and case-fatality rate.^3^ EV71 is regarded as one of the most dangerous neurotropic enteroviruses in the absence of vaccines and effective antiviral therapy.^4^ EV71 infections may be complicated by severe neurological complications or fatal pulmonary edema. EV71 is a highly neurotropic virus and the brainstem is its most likely major target. The mechanism of pulmonary edema may be related to increased pulmonary vascular permeability caused by brainstem lesions and/or systemic inflammatory response rather than increased pulmonary capillary hydrostatic pressure. The neonate in the present case, who was infected with EV71, suffered from severe HFMD complicated with both brainstem encephalitis and pulmonary edema.

The bad prognosis and mortality in few cases are associated with acute onset and rapid deterioration or severe illness associated with encephalitis, brainstem encephalitis, encephalomyelitis, pulmonary edema, pulmonary hemorrhage and circulatory failure.^1^ The risk factors associated with severe HFMD are young male children (≤2 years old), atypical physical findings (tachycardia, tachypnea, hypotension, hypertension, bleeding in the gastrointestinal tract, neurological deficits and pulmonary edema), a raised total leukocyte count, vomiting, absence of oral lesions and hyperglycemia.^5^ This patient was classified with severe HFMD because of the existence of several risk factors, including neonate, brainstem encephalitis, pulmonary edema, shock, stress hyperglycemia, a raised total leukocyte count and the absence of oral lesions.

## CONCLUSIONS

Severe HFMD in neonates is extremely rare and may be easily misdiagnosed. Additional clues in the medical history and a careful physical examination are very important in making an early, prompt diagnosis. Although there is no effective antiviral therapy for HFMD, a good supportive therapy is likely to result in complete recovery.

## Authors Contribution:

**Shi-Jie Guo** drafted the manuscript; **Dong-Xuan Wang** collected all data; **Chun-Lai Dai** data analysis; **Hui Wu** revised and approved the final manuscript.

## Notes

***Funding: ***None.

## References

[1] Zhang Y, Zhu Z, Yang W, Ren J, Tan X, Wang Y (2010). An emerging recombinant human enterovirus 71 responsible for the 2008 outbreak of hand foot and mouth disease in Fuyang city of China. Virol J.

[2] Rabenau HF, Richter M, Doerr HW (2010). Hand, foot and mouth disease: seroprevalence of Coxsackie A16 and Enterovirus 71 in Germany. Med Microbiol Immunol.

[3] Tran CB, Nguyen HT, Phan HT, Tran NV, Wills B, Farrar J (2011). The seroprevalence and seroincidence of enterovirus 71 infection in infants and children in Ho Chi Minh City. Viet Nam.

[4] Modlin JF (2007). Enterovirus deja vu. N Engl J Med.

[5] Ooi MH, Wong SC, Mohan A, Podin Y, Perera D, Clear D (2009). Identification and validation of clinical predictors for the risk of neurological involvement in children with hand, foot, and mouth disease in Sarawak. BMC Infect Dis.

